# Sofosbuvir Activates EGFR-Dependent Pathways in Hepatoma Cells with Implications for Liver-Related Pathological Processes

**DOI:** 10.3390/cells9041003

**Published:** 2020-04-17

**Authors:** Denisa Bojkova, Sandra Westhaus, Rui Costa, Lejla Timmer, Nora Funkenberg, Marek Korencak, Hendrik Streeck, Florian Vondran, Ruth Broering, Stefan Heinrichs, Karl S Lang, Sandra Ciesek

**Affiliations:** 1 Institute of Virology, University Hospital Essen, University Duisburg-Essen, 45147 Essen, Germany; denisa.bojkova@kgu.de (D.B.); sandra.westhaus@kgu.de (S.W.); rcosta@sund.ku.dk (R.C.); lejla.timmer@uk-essen.de (L.T.); nora.funkenberg@googlemail.com (N.F.); 2 Institute of Medical Virology, University Hospital, Goethe University Frankfurt am Main, 60590 Frankfurt, Germany; 3 Institute for HIV research, University Hospital Essen, University Duisburg-Essen, 45147 Essen, Germany; marek.korencak@uk-essen.de (M.K.); hendrik.streeck@uk-essen.de (H.S.); 4 Clinic for General, Abdominal and Transplant Surgery, Hannover Medical School, 30625 Hannover, Germany; vondran.florian@mh-hannover.de; 5 German Center for Infection Research (DZIF), 45147 Essen, Germany; 6 Department of Gastroenterology and Hepatology, University Hospital Essen, University Duisburg-Essen, 45147 Essen, Germany; ruth.broering@uni-due.de; 7 Institute for Transfusion Medicine, University Hospital Essen, University Duisburg-Essen, 45147 Essen, Germany; stefan.heinrichs@uk-essen.de; 8 Institute of Immunology, University Hospital Essen, University Duisburg-Essen, 45147 Essen, Germany; KarlSebastian.Lang@uk-essen.de

**Keywords:** direct-acting antivirals, HCV, HCC recurrence, nucleotide analogue, EGFR pathway

## Abstract

Direct acting antivirals (DAAs) revolutionized the therapy of chronic hepatitis C infection. However, unexpected high recurrence rates of hepatocellular carcinoma (HCC) after DAA treatment became an issue in patients with advanced cirrhosis and fibrosis. In this study, we aimed to investigate an impact of DAA treatment on the molecular changes related to HCC development and progression in hepatoma cell lines and primary human hepatocytes. We found that treatment with sofosbuvir (SOF), a backbone of DAA therapy, caused an increase in EGFR expression and phosphorylation. As a result, enhanced translocation of EGFR into the nucleus and transactivation of factors associated with cell cycle progression, B-MYB and Cyclin D1, was detected. Serine/threonine kinase profiling identified additional pathways, especially the MAPK pathway, also activated during SOF treatment. Importantly, the blocking of EGFR kinase activity by erlotinib during SOF treatment prevented all downstream events. Altogether, our findings suggest that SOF may have an impact on pathological processes in the liver via the induction of EGFR signaling. Notably, zidovudine, another nucleoside analogue, exerted a similar cell phenotype, suggesting that the observed effects may be induced by additional members of this drug class.

## 1. Introduction

With approximately 71 million chronically infected patients, hepatitis C virus (HCV) represents one of the leading etiologies for the development of hepatocellular carcinoma (HCC) worldwide [1,2]. Recently introduced direct acting antivirals (DAAs) have dramatically improved the treatment of chronic HCV infection. Nowadays, DAAs allow a sustained virological response to be achieved in more than 95% of patients without major side effects [3,4,5]. Even though these new drugs represent a huge breakthrough for a majority of chronically HCV-infected patients, the benefit of interferon-(IFN)-free therapies for a subset of patients has recently been questioned by several groups. Two studies showed an increase in the recurrence rates of HCC (27% and 29%) after DAA treatment in patients who had been successfully treated for HCC prior to the start of DAA therapy and were disease free for different periods of time [6,7]. Moreover, the recurrent tumors exhibited signs of microvascular invasion and were characterized by a more aggressive phenotype with faster progression to advanced stages [8]. Further studies have confirmed the increase in the recurrence/occurrence of HCC after DAA treatment, whereas others have refuted these results [9,10,11,12,13].

Despite these contradictory reports, several mechanisms for the high rate of tumor relapses and de novo tumors after DAA therapy have been proposed. A decrease of inflammation signals followed by a reduction of immune surveillance could allow tumor clones to progress without immediate recognition and elimination by the immune system [7]. Indeed, several research groups showed that the clearance of HCV with DAA treatment changed the innate immunity [14,15,16]. Other studies indicated a potential effect of IFN-free therapy on angiogenesis [17,18]. They observed an increase in the serum concentrations of vascular endothelial growth factor (VEGF) and angiopoietin-2, growth factors responsible for vascular remodeling in tumors, during DAA treatment and this increase correlated with a higher risk of HCC relapse and de novo occurrence [17,18]. All these studies provide a possible rationale for the involvement of DAA treatment in the modification of the local immune status, cytokine signaling network, and pro-angiogenesis molecules. However, the underlying modified cellular pathways have not yet been elucidated.

In light of these results, we aimed to investigate how DAAs modulate relevant molecular pathways and protein expression involved in liver pathological processes in liver-derived cell lines. Our analysis revealed an altered cell phenotype following sofosbuvir treatment, which was characterized by changes in the cell cycle distribution, expression of cell cycle-regulating factors, and proliferation. Further investigation identified the activation of epidermal growth factor receptor (EGFR) signaling by its phosphorylation and translocation into the nucleus as a driver of these alterations.

## 2. Materials and Methods

### 2.1. Cell Culture and Compounds

HepG2, HuH-6, Huh-7, and HEK293 cells were maintained in Dulbecco’s modified Eagle medium (DMEM, Thermo Fisher Scientific, Schwerte, Germany) containing 10% (*v/v*) fetal bovine serum (Biochrom, Cambridge, UK) and 10,000 U penicillin/streptomycin, 1% (*v/v*) l-glutamine, and 1% (*v/v*) non-essential amino-acids (Thermo Fisher Scientific, Schwerte, Germany). Primary human hepatocytes (PHHs) were isolated as previously described [19,20]. PHHs were maintained in William’s Medium E (PAN Biotech, Aidenbach, Germany) containing 10% (*v/v*) fetal bovine serum (Biochrom, Cambridge, UK) and 10,000 U penicillin/streptomycin, 1% (*v/v*) l-glutamine, 1% (*v/v*) non-essential amino-acids, 5mmol/L Hepes (Thermo Fisher Scientific, Schwerte, Germany), 2% (*v/v*) dimethyl sulfoxide (DMSO, Roth, Karlsruhe, Germany), 5 µg/mL insulin, and 0.05 mmol/L hydrocortisone (Sigma Aldrich, Munich, Germany). Sofosbuvir, daclatasvir, simeprevir, erlotinib, doramapimod, zidovudine, and tenofovir (Selleckchem, Munich, Germany) were dissolved in DMSO (Roth, Karlsruhe, Germany) and diluted in DMEM at the final concentration depicted in each figure.

### 2.2. Cell Cycle Analysis by DNA Staining

Cells were fixed and permeabilized with BD Cytofix/Cytoperm™ (BD Bioscience, San Jose, CA, USA) and stained with CytoPhase™ Violet (BioLegend, London, UK). DNA content was measured by flow cytometry using BD FACS Canto II (Backman Coulter, Krefeld, Germany). Cell cycle distribution was determined by FCS Express 6 (De Novo Software, Glendale, CA, USA).

### 2.3. Measurement of Apoptosis, Proliferation, and Cytotoxicity

Apoptosis was detected through double staining of the membrane alteration (phosphatidylserine flip) with Annexin V and live versus dead status of cells with Zombie Violet according to the manufacturer’s protocol (BioLegend, London, UK). Flow cytometry using BD FACS Canto II (Backman Coulter, Krefeld, Germany) was applied to define the Annexin V+/Zombie Violet- population as early apoptotic cells and Annexin V+/Zombie Violet+ as late apoptotic cells. Cell proliferation was assayed by trypan blue exclusion of cells counted by phase microscopy. Cytotoxicity of different DAAs was determined using the Rotitest^®^ Vital (Roth, Karlsruhe, Germany) according to the manufacturer’s instructions.

### 2.4. Immunoblot Analysis

Whole cell lysates were prepared with M-PER™ Mammalian Protein Extraction Reagent containing protease and phosphatase inhibition cocktail (Thermo Fisher Scientific, Schwerte, Germany). Nuclear and cytoplasm fractions were obtained with NE-PER™ Nuclear and Cytoplasmic Extraction Reagent (Thermo Fisher Scientific, Schwerte, Germany). Proteins were separated by SDS-PAGE (Bio-Rad, Munich, Germany) and transferred to PVDF membrane. Immunoblot analysis was performed using the following antibodies: Anti-B-MYB, anti-EGFR, anti-pEGFR, anti-CyclinD1, anti-β-tubulin, anti-LaminB1 (Abcam, Cambridge, UK), p38, p-p38 (CST, Frankfurt am Main, Germany), and anti-β-actin (Sigma-Aldrich, Munich, Germany). Proteins were visualized using peroxidase-coupled secondary antibodies anti-rabbit (Sigma-Aldrich, Munich, Germany) or anti-mouse (Dianova, Hamburg, Germany) and an enhanced chemiluminescence system (GE Healthcare, Buckinghamshire, UK). Immunoblots were analyzed by Gel analyzer plugin in ImageJ 1.50i and the values of target proteins were normalized to a housekeeping gene.

### 2.5. Serine/Threonine Kinase Profiling

Serine/threonine kinase (STK) activity profiles were determined with the serine/threonine PamChip^®^ peptide microarray system (PamGene International B.V., BJ’s-Hertogenbosch, Netherlands). Each microarray contains a porous matrix probed with covalently attached 144 STK-specific conserved peptides of phosphomotifs, enabling constant flow-through of the lysates containing activated or inactivated kinases in the presence of ATP, and thus facilitating the phosphorylation of peptides. Subsequently, the phosphorylation is detected using fluorescently labelled antibodies. Preparation of the samples, phosphorylation measurement, and data analysis were performed according to the manufacturer´s protocol (www.pamgene.com). Briefly, cells were lysed with M-PER™ containing protease and phosphatase inhibition cocktail and stored at −80 °C till the measurement. The measurement was performed on a PamStation^®^12 system utilizing the evolve protocol (1300STKlysv09.PS12Protocol, PamGene). For the detection, 0.5 µg of protein lysate was applied. Quantification of the peptides’ phosphorylation was conducted using Bionavigator software (PamGene). Since one peptide can be phosphorylated by several kinases and kinases can usually phosphorylate several peptides, PamGene developed a tool, Upstream kinase analysis, to identify the most likely activated kinases. The Upstream kinase analysis is based on the comparison of phosphorylated peptides on an array with databases of documented interactions, such as HPRD, PhosphoSitePlus as well as the in-silico predictions database PhosphoNET. Based on a specificity and significance score, the analysis classifies kinases according to a median final score (MFS) and median kinase statistics (MKS). The resulting list of kinases is based on the final score [MFS+MKS]. Final scores were clustered using the heatmaps2 function of the gplots package of the R suite. Briefly, distances were calculated using Pearson correlation and cell line clustering was calculated via UPGMA. Scripts are available upon request.

### 2.6. Statistics

All experiments were performed under similar conditions. The respective number of independent experiments is depicted in each figure legend. Prism 6 software (GraphPad software, San Diego, CA, USA) was used to plot the graphical representation and to perform statistical analysis. Statistical significance was calculated by one-way ANOVA, two-way ANOVA, or an unpaired/paired *t*-test as described in the figure legends (ns—not significant, * *p* < 0.05, ** *p* < 0.01, *** *p* < 0.005).

## 3. Results

### 3.1. Cell Cycle Distribution after DAA Treatment in Hepatoma Cell Lines

**Figure 1:**
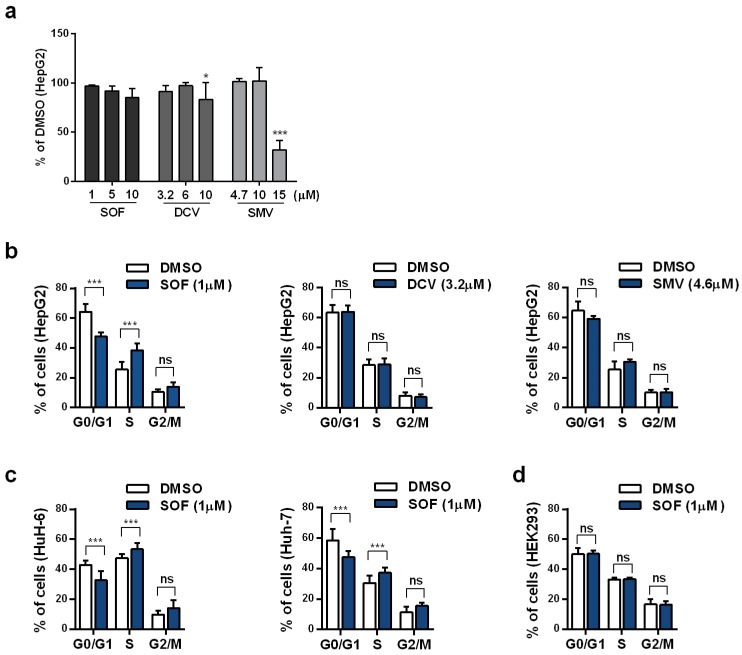
Cell cycle distribution after DAA treatment. (**a**) Cytotoxicity of an increasing concentration of each DAA in HepG2 cells was detected by Rotitest^®^ Vital. Bar graph displays the absorbance as a fold change in relation to DMSO. Cell cycle distribution was evaluated by flow cytometric analysis of DNA content in HepG2 cells treated with SOF, DCV, or SMV (**b**); HuH-6 and Huh-7 cells (**c**); and HEK293 cells (**d**) treated with SOF for four consecutive days. Data are displayed as the percentage of cells in each phase. All shown data represent mean + s.d. from three independent experiments. Statistical significance was determined through two-way ANOVA (**a**–**d**). ns: not significant; * *p* ≤ 0.05; *** *p* ≤ 0.005.

First, we tested whether therapeutic concentrations of different DAAs [21,22,23] exhibited cytotoxicity in our hepatoma cell model, HepG2 cells. For that purpose, HepG2 cells were treated for four consecutive days with DAAs from each major drug class: Sofosbuvir (SOF, NS5B polymerase inhibitor), daclatasvir (DCV, NS5A protein inhibitor), and simeprevir (SMV, NS3-4A protease inhibitor). Drug-containing cell culture medium was replaced daily. The investigated drug concentrations, which included the maximum concentrations of each drug detected in patient plasma, did not cause toxic effects in hepatoma cells (Figure 1a).

Next, we tested if different DAAs have any impact on the cell cycle distribution of hepatoma cells. As shown in Figure 1b, SOF treatment led to a significant decrease in the percentage of cells in G0/G1 phase from 64.2% to 47.6% while the percentage of cells in S and G2/M phase increased from 25.5% to 38.4% and from 10.3% to 14.0%, respectively. The same effect of SOF on the cell cycle was confirmed in two additional hepatoma cell lines, HuH-6 and Huh-7 (Figure 1c). No effect on the cell cycle distribution by DCV or SMV was detected. SOF as a prodrug requires metabolic activation to its active triphosphate (TP) form to exhibit its effect [21]. In this context, hepatocytes possess the strongest ability to convert SOF to its active metabolite whereas non-hepatic cells do not support this conversion [24]. Here, we confirmed that in non-hepatic cells, HEK293 (Figure 1d), SOF treatment did not detectably alter the cell cycle distribution.

### 3.2. Sofosbuvir Induces Pro-Survival Changes in Hepatoma Cells

An increase in the proportion of cells in S phase following SOF treatment could suggest DNA damage with ongoing DNA repair mechanisms. SOF is an uridine nucleotide analogue (NA) able to incorporate into the HCV RNA chain and thereby block viral replication [21]. Interestingly, a number of HCV NAs failed in phase II mainly due to off-target effects impairing mitochondrial protein synthesis [25]. Crucially, our monitoring of mitochondrial respiration during SOF treatment did not reveal any impairment (Figure S1a,c).

**Figure 2:**
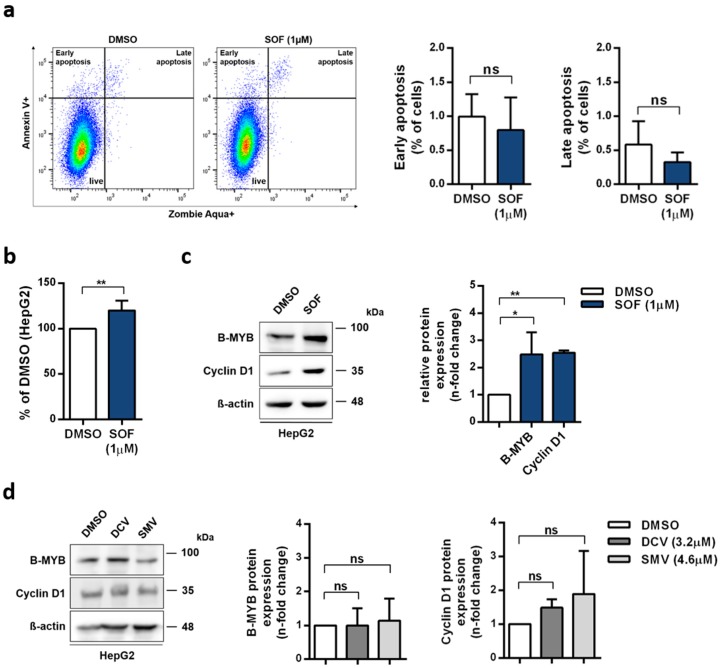
Impact of SOF treatment on cell cycle progression (**a**) Proportion of apoptotic cells was determined with Annexin V and live/dead cells staining in HepG2 cells incubated with SOF at day five. (**b**) Proliferation rates of SOF-treated cells were evaluated by trypan blue exclusion and displayed in relation to the vehicle control DMSO. (**c**,**d**) B-MYB and Cyclin D1 protein expression after SOF (**c**) and DCV or SMV (**d**) treatment were analyzed by immunoblot. One representative immunoblot is shown. Bar graph displays relative protein expression as fold change in relation to DMSO. All shown data represent mean + s.d. from three (a, b, c B-MYB, d) and two (d CyclinD1) independent experiments. Statistical significance was determined through one-way ANOVA (**c**,**d**) and unpaired *t*-test (**a**,**b**). ns: not significant; * *p* ≤ 0.05; ** *p* ≤ 0.01.

As a response to DNA damage, cells are prompted to apoptosis or survival [26]. In order to elucidate the additional molecular events accompanying cell cycle distribution changes caused by SOF, we investigated the induction of apoptosis (Figure 2a) and proliferation rates (Figure 2b). No increase in the proportion of apoptotic cells was detected. Whereas, the proliferation rate after SOF therapy was higher compared to the vehicle control. Together, these data suggest that cells were directed towards survival. Interestingly, the rates of glycolysis and glycolytic capacity (Figure S1b,d) had an upward trend accompanying rising concentrations of SOF, which might be a reaction to an increased demand of metabolites resulting from enhanced proliferation. Additionally, SOF (Figure S2a) had no effect on the proliferation of HEK293 cells, which further points to the active triphosphate form of SOF as the driver of alterations in hepatoma cells. DCV and SMV did not alter the proliferation rates (Figure S2b).

Next, we evaluated the expression of cell cycle-regulating factors, Myb-related protein B (B-MYB) and Cyclin D1, which are responsible for G1/S transition [27,28]. Additionally, B-MYB is also required for the expression of late cell cycle genes [28]. As depicted in Figure 2c, both proteins were increased in SOF-treated cells. In contrast, the B-MYB and Cyclin D1 protein levels did not change after DCV or SMV treatment (Figure 2d). Moreover, SOF had no effect on B-MYB expression in HEK293 cells (Figure S2c).

In summary, SOF, but not DCV or SMV, altered the expression of cell cycle-regulating factors, accompanied by a shift in the cell cycle distribution. Moreover, an observed increase in proliferation and no signs of apoptosis indicate a pro-survival reprogramming of the cells treated with SOF. We also confirmed the obligatory SOF metabolic activation in the induction of cell fate reprogramming given that the non-hepatic cell line HEK293 did not support molecular changes resulting from exposure to SOF.

### 3.3. SOF Induces Activation of EGFR in Hepatoma Cells

**Figure 3:**
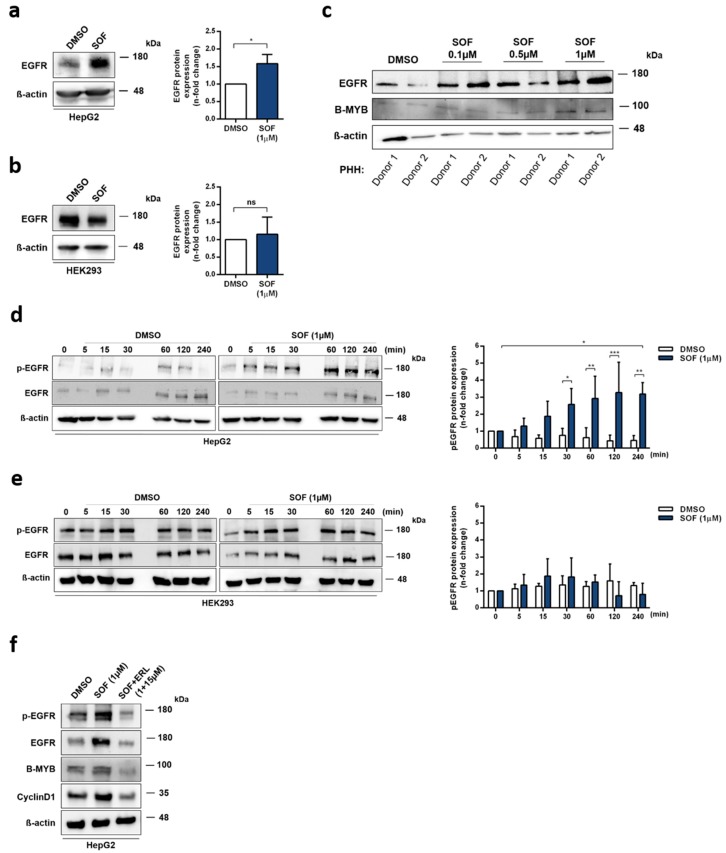
Sofosbuvir increases the expression and activation of EGFR. (**a**,**b**) EGFR protein levels after SOF treatment were identified by immunoblot analysis. One representative immunoblot is displayed. EGFR expression is presented in the bar graphs as a fold change relative to the DMSO control (mean + s.d. from three biological replicates). (**c**) PHHs were incubated with rising concentrations of SOF for four days. EGFR and B-MYB protein levels were evaluated by immunoblot analysis. One representative immunoblot of two independent experiment is displayed. (**d**) HepG2 and (**e**) HEK-293 cells were starved for 24 h prior to SOF treatment. Protein levels of the phosphorylated form of EGFR (pEGFR) were assessed at the depicted time points by immunoblot analysis. One representative immunoblot is presented. pEGFR expression is shown as a fold change in relation to time point 0 (mean + s.d. from three biological replicates). (**f**) EGFR, pEGFR, B-MYB, and Cyclin D1 protein levels after treatment with SOF and SOF in combination with the EGFR inhibitor, erlotinib (ERL), were identified by immunoblot analysis. One representative immunoblot of two independent experiments is shown. Statistical significance was determined through an unpaired *t*-test (**a**,**b**), paired *t*-test (d, time point 0 vs. 240 min), and two-way ANOVA (**d**,**e**). ns: not significant; * *p* ≤ 0.05; ** *p* ≤ 0.01; *** *p* ≤ 0.005.

The expression of cell cycle-regulating factors like B-MYB and Cyclin D1 is strongly regulated at many different levels. Interestingly, one common upstream regulator of these two proteins is epidermal growth factor receptor (EGFR) [29,30]. On this account, we aimed to examine whether SOF treatment also influences EGFR expression. The SOF-treated HepG2 cells (Figure 3a) displayed an increased expression of the EGFR protein, which, in contrast, was not observed in HEK293 cells (Figure 3b). Additionally, we examined whether the elevated expression of EGFR and its downstream target B-MYB also occurs in primary human hepatocytes (PHHs) after SOF treatment. As demonstrated in Figure 3c, both EGFR and B-MYB protein levels increased in the presence of SOF.

Transactivation of B-MYB and Cyclin D1 expression driven by EGFR activation requires, as a first step, the phosphorylation of EGFR [29,30]. To verify that EGFR is increasingly phosphorylated in SOF-treated cells, HepG2 and HEK293 cells were starved for 24 h and subsequently treated with SOF or the vehicle control for different periods of time (0–240min). Figure 3d documents a time-dependent increase in the phosphorylation of EGFR after SOF treatment whereas the DMSO control did not exhibit increased phosphorylation. In HEK293 cells (Figure 3e), SOF had no effect on EGFR phosphorylation.

To further study the functional relevance of EGFR activation after SOF treatment, we utilized an EGFR inhibitor, erlotinib (ERL), together with SOF. As shown in Figure 3f, the addition of ERL led to a reduced expression of EGFR, pEGFR, B-MYB, and Cyclin D1. Moreover, we confirmed that the presence of ERL induced apoptosis (Figure S3a) and prevented an SOF-related increase in proliferation (Figure S3b).

In summary, SOF specifically altered the expression and activation of EGFR in liver-derived cells, which in turn led to an increased expression of its downstream targets. These events could be prevented by the employment of an EGFR inhibitor during SOF treatment.

### 3.4. Activity-Based Kinases Profiling in Hepatoma Cells Reveals Pathways Activated by SOF Treatment

**Figure 4:**
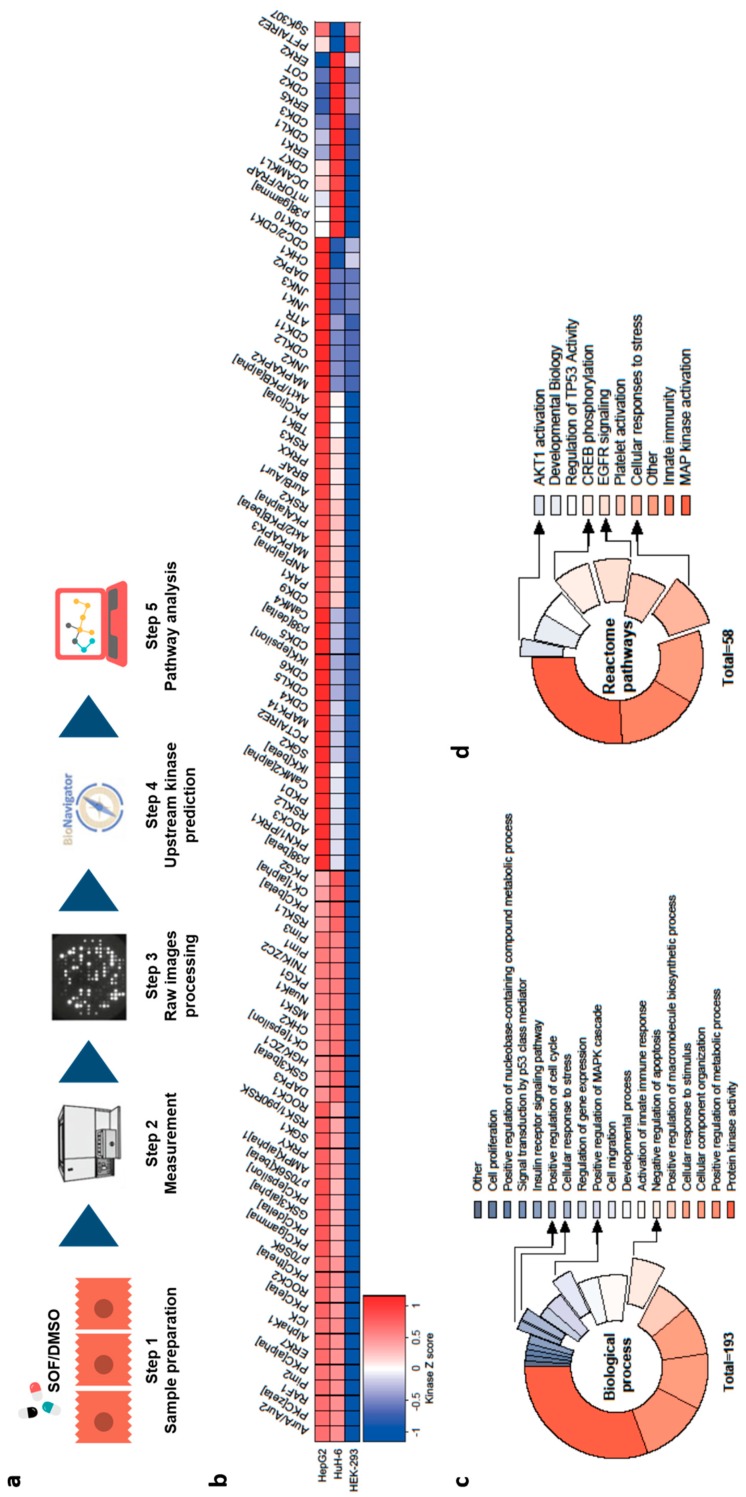
Kinases activation profiling after SOF treatment. (**a**) Representation of STK kinase activation profiling. (**b**) Heatmap of kinases with the highest probability of activation in HepG2, HuH-6, and HEK293 cells after SOF treatment sorted from high (red) to low (blue) kinase Z score. (**c**,**d**) Pathway analysis of top activated kinases in HepG2 cells (final score >2) based on their enrichment in specific biological processes (GeneGo, corrected *p* value < 10^−4^) (**c**) or reactome pathways (STRING, false discovery rate < 10^−4^) (**d**). Displayed data represent three independent experiments.

Phosphorylation of EGFR as a receptor tyrosine kinase leads to a downstream signal transduction, resulting in the activation of a wide range of pathways and the expression of a significant number of genes. Since the majority of phosphoproteome results from serine/threonine phosphorylation [31], we aimed to identify serine/threonine kinases (STKs) activated upon SOF treatment. As depicted in Figure 4a, we utilized high-throughput STK PamChip^®^ array based on the measurement of peptide phosphorylation. Figure 4b displays the STK profiles of the most likely activated kinases after SOF treatment versus the control in HepG2, HuH-6, and HEK293 cells. The exact value of the final score for each kinase is depicted in Table S1. We could clearly observe the activation of several STK in the hepatoma cell lines HepG2 and HuH-6, whereas in non-hepatic HEK293 cells, SOF treatment had no impact on STK activation. This further highlights the active form of SOF as being responsible for the phenotype alteration observed in hepatoma cells.

To further reveal the interactions between activated STK in HepG2 cells and analyze their influence on biological processes, the highest ranked kinases (final score >2) were subjected to pathway analysis by GeneGo (Figure 4c) and STRING v11 (Figure 4d). Expectedly, the most enriched pathway regarding biological processes was protein kinase activity. Biological process terms, such as the cellular response to stress, positive regulation of the cell cycle, and negative regulation of apoptosis, were highly enriched with the activated kinase list as input. Additionally, we observed enrichment of pathways, such as the mitogen-activated protein kinase (MAPK) cascade, Akt1 activation, and cAMP response element-binding protein (CREB) phosphorylation, which are downstream in the EGFR signaling network. On this account, we evaluated the expression of the phosphorylated form of Akt, c-Raf, and MEK1/2 (Figure S4). We could show an increase in the phosphorylation of both Akt and MEK1/2 after SOF treatment, which further confirms the activation of EGFR downstream signaling after SOF treatment.

Taken together, high-throughput STK profiling after SOF treatment in different cell lines revealed the activation of several signaling pathways downstream of EGFR and further confirmed the role of the active SOF TP form in the establishment of an altered cell phenotype.

### 3.5. Regulation and Nuclear Translocation of SOF-Activated EGFR

**Figure 5:**
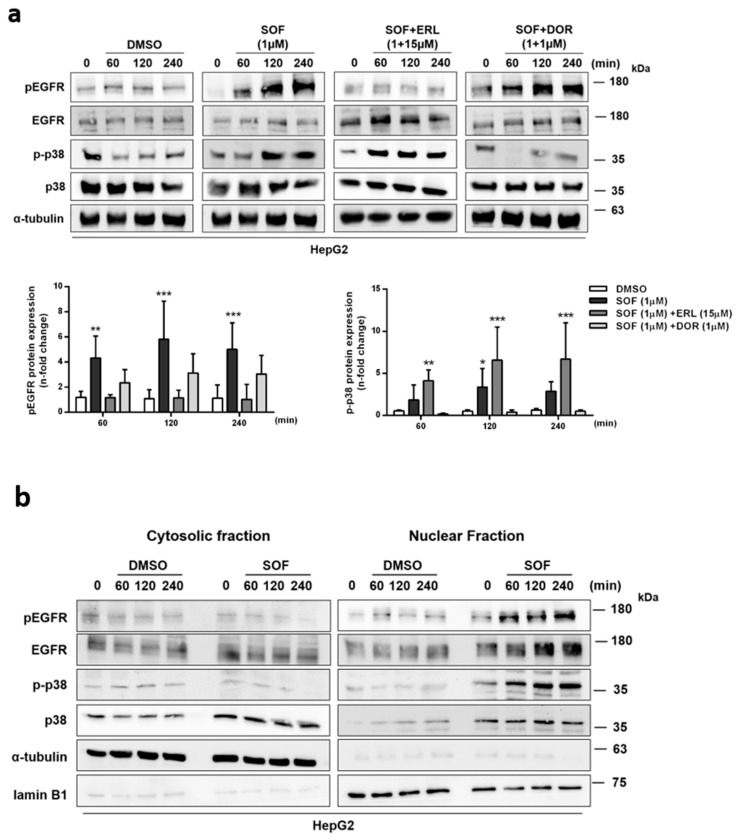
Regulation of EGFR phosphorylation and nuclear translocation during SOF treatment. (**a**) HepG2 cells were starved for 24 h and subsequently treated with SOF alone or in combination with pEGFR inhibitor (ERL) or p-p38 inhibitor (DOR). The protein level of target proteins was assessed at depicted time points by immunoblot analysis. One representative immunoblot is presented. Bar graphs display the relative quantification of pEGFR and p-p38 shown as fold change in relation to time point 0 (mean + s.d. from three independent experiments). Statistical significance was determined through two-way ANOVA (DMSO vs. treatment). * *p* ≤ 0.05; ** *p* ≤ 0.01; *** *p* ≤ 0.005. (**b**) The expression of target proteins in the cytoplasm and nucleus after SOF treatment at different time points was detected by immunoblot an lysis. One representative immunoblot of three (EGFR, pEGFR) and two (p-p38, p38) independent experiments is shown.

In the context of downstream EGFR signaling, positive regulation of the MAPK cascade appeared to be activated after SOF treatment. Interestingly, one member of this cascade, p38 (MAPK14), is also able to act upstream of EGFR and initiate its phosphorylation and internalization as a response to stress [32]. This can be prevented by utilizing p38 inhibitors [33]. Therefore, we were interested if SOF acts as a stress inducer recognized by p38, which in consequence activates EGFR. On this account, we applied both a p38 inhibitor, doramapimod (DOR), and an EGFR inhibitor, erlotinib (ERL), during SOF treatment and evaluated the activation of both p38 and EGFR (Figure 5a).

We could confirm that p38 is indeed activated during SOF treatment. The addition of ERL to the SOF treatment led to the inhibition of EGFR phosphorylation. However, p38 phosphorylation was still induced, which could be explained by the pro-apoptotic effect of ERL [34]. DOR in combination with SOF effectively blocked p38 phosphorylation. In contrast, no inhibitory effect on EGFR phosphorylation was observed, but the EGFR phosphorylation exhibited rather an increasing tendency over time just as was observed with the SOF treatment only. These results suggest that p38 activation is not crucial for the induction of EGFR phosphorylation observed during SOF treatment.

One interesting feature of EGFR in addition to its traditional well-described signaling pathway is its ability to translocate into the nucleus, where it is responsible for transcriptional regulation of genes involved in cell cycle regulation, proliferation, and DNA repair [35]. Additional kinases, as well as p38, were reported to similarly undergo nuclear translocation [36]. To investigate if EGFR and p38 translocation also occur during SOF treatment, we evaluated the level of both proteins in the cytoplasm and the nucleus. As demonstrated in Figure 5b, the levels of the phosphorylated forms of both proteins were increased in the nuclear fraction after exposure to SOF.

In summary, our data suggest a p38-independent mechanism of EGFR activation during SOF treatment. We also confirmed EGFR nuclear translocation upon SOF treatment. Additionally, the same event was also observed for p38.

### 3.6. Role of Other Nucleoside Analogues in EGFR Activation

**Figure 6:**
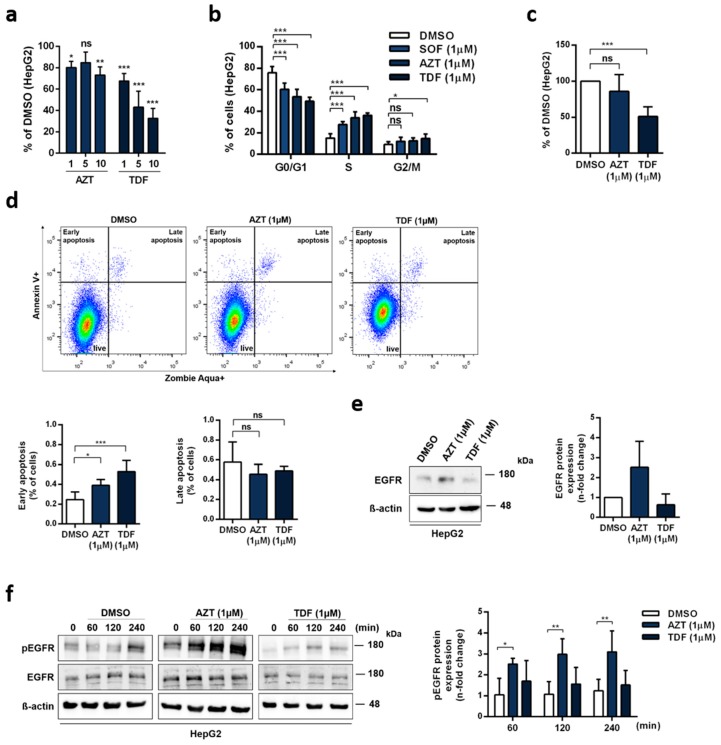
Effect of different nucleotide analogues on alteration in the cell phenotype and activation of EGFR. (**a**) Cytotoxicity of zidovudine (AZT) and tenofovir (TDF) in HepG2 cells was detected by Rotitest^®^ Vital. Bar graph displays absorbance as a fold change in relation to DMSO. (**b**) Cell cycle analysis of HepG2 cells treated with AZT and TDF. (**c**) Apoptosis induction was evaluated with Annexin V and live/dead cell staining by flow cytometry. (**d**) Proliferation rates were determined by trypan blue exclusion and displayed in relation to DMSO. (**e**) EGFR expression after four days of continuous treatment with AZT and TDF. One representative immunoblot is shown. Bar graph presents relative quantification of EGFR as a fold change compared to DMSO. (**f**) Activation of EGFR after AZT and TDF therapy was evaluated by immunoblot analysis at different time points. One representative immunoblot is depicted. pEGFR expression is shown as a fold change in relation to time point 0. All graphs present mean + s.d. from three independent experiments. Statistical significance was determined through one-way ANOVA (**a**,**c**,**d**) and two-way ANOVA (**b**,**f**). ns: not significant; * *p* ≤ 0.05; ** *p* ≤ 0.01; *** *p* ≤ 0.005.

Since SOF belongs to a broad group of nucleotide analogues (NAs), we further investigated if the altered phenotype in hepatoma cells can also be detected with other nucleoside analogues. On this account, we utilized zidovudine (AZT) and tenofovir (TDF), which are both widely used in clinical practice. As shown in Figure 6a, both drugs decreased the cell viability of HepG2 cells even at the lowest concentration. This was also mirrored in the cell cycle distribution (Figure 6b), apoptosis induction (Figure 6c), and proliferation (Figure 6d). Compared to SOF, both AZT and TDF displayed a stronger effect on the cell cycle distribution, with a reduction of cells in G0/G1 phase and an increase in S phase. In case of TDF, an increase in G2/M phase was also observed. Additionally, both AZT and TDF increased the rates of early apoptosis. In the case of TDF, this observation also correlates with reduced proliferation rates. Interestingly, we observed that AZT but not TDF led to an increased expression of EGFR (Figure 6e). As depicted in Figure 6f, this was in line with the high levels of EGFR phosphorylation induced upon AZT treatment.

Altogether, we showed that NAs were able to cause alteration of the cell cycle in hepatoma cells; however, the outcome differed between them. Whereas AZT induced changes comparable to SOF, increased expression, and phosphorylation of EGFR, TDF manifested its effect on cells by an induction of apoptosis and a decrease in cell proliferation without any signs of EGFR activation.

## 4. Discussion

The introduction of a novel highly effective therapy with DAA able to clear HCV infection in over 95% of patients has raised expectations of reducing liver cancer in chronically HCV-infected patients [3,4]. Despite data supporting an improvement of liver function after DAA therapy [5], several other reports presented findings questioning the impact of IFN-free treatment on the risk of HCC development [6,7]. These studies have reported a higher number of tumor relapses in patients who underwent successful HCC treatment and were disease free before starting DAA therapy than expected [6,7,37]. In our study, we hypothesized that DAA treatment might introduce certain cellular signaling processes facilitating tumor progression in individual patients with pre-existing pro-oncogenic changes in liver tissue. The screening of phenotype changes among different DAA demonstrated an SOF-driven pro-survival alteration, which was not observed with the two other drugs, DCV and SMV.

SOF is a prodrug of an uridine nucleotide analogue with pan-genotypic activity, which is used as a backbone in DAA-based therapies [21]. NAs exert their cytotoxicity mainly by interfering with host DNA replication, resulting in a DNA damage response, which at a molecular level decides the cell fate: Survival or apoptosis. The outcome is based on the imbalance between pro-survival and pro-apoptotic factors [38]. An observed increase in proliferation rates and no induction of apoptosis after SOF treatment indicated that the cells were destined to survive and proliferate rather than initiate apoptosis. Other evidence for a pro-survival reprogramming of the hepatoma cells was the elevated expression of B-MYB and Cyclin D1 after SOF exposure. Both proteins are required for progression through the cell cycle, and their responsibility for the high proliferation capacity of tumor cells in multiple cancers is well documented [39,40,41]. Together with their pro-survival role during the DNA damage response [40,42], it further suggests an active involvement in cell survival following treatment with SOF.

NAs play an important role in antiviral therapies because of their potency and high barrier to resistance. Interestingly, a number of HCV NAs failed in phase II mainly due to off-target effects impairing mitochondrial protein synthesis [25]. However, mitochondrial respiration did not appear to be impaired by SOF. Based on our results and previous findings, SOF also seems to be a poor substrate for mitochondrial RNA polymerase [43]. Therefore, off-target incorporation of SOF affecting mitochondrial respiration seems unlikely. The question of how/if SOF induces a DNA damage response remains open. Interestingly, increasing SOF concentrations were associated with a non-significant upward trend in glycolysis and glycolytic capacity, which may reflect an increased demand of metabolites in response to enhanced proliferation or positive feedback in nucleotide anabolism, given both glycose-6-phosphate and phosphoenolpyruvate are precursors of nucleotide synthesis.

EGFR is a very well-studied receptor tyrosine kinase, which mediates extremely complex signal transduction [31]. Besides the traditional cytoplasmic pathway leading to the activation of pathways responsible for cell cycle progression, proliferation, and resistance to apoptosis, EGFR possesses the ability to translocate into the nucleus in response to ligand stimulation or stress inducers [29,30,44]. Based on the origin of the stimulation, nuclear EGFR acts as a transcription enhancer of a subset of genes, of which B-MYB and Cyclin D1 exhibited elevated expression after exposure to SOF. On this account, we first confirmed the increase in the phosphorylation of EGFR in response to SOF stimulation, which in turn led to an enhanced expression of EGFR after a four-day exposure to the drug. This is consistent with previous findings showing that prolonged activation of EGFR enhances EGFR transcription through proteins downstream in the EGFR signaling pathway [45,46]. Next, by introducing an EGFR inhibitor during SOF treatment, we reversed the pro-survival outcome mediated by EGFR activation and directed cells towards apoptosis. By detecting an increase of phosphorylated EGFR in the nucleus, we validated our proposed model: As a result of the active TP form of SOF in hepatoma cells, EGFR is activated and translocated into the nucleus, where it enhances the transcription of pro-survival genes.

Phosphorylation of EGFR results in a signal transduction network with diverse outcomes, where none of the signaling pathways act separately but in a highly inter-linked way. Therefore, we assumed that the activation of EGFR during SOF treatment with its subsequent nuclear translocation is not a separate event, but it can involve an entire signaling network. To untangle this network, we utilized global kinase activity profiling. Based on the identified kinome activation, we elucidated the biological processes and reactome pathways activated during SOF exposure. We confirmed the activation of biological processes as positive regulation of the cell cycle, cell proliferation, and negative regulation of apoptosis. In addition, we observed the activation of several pathways located downstream of EGFR signaling, such as AKT1 activation and CREB phosphorylation, whose role in promoting cell survival by directly inactivating components of the cell death machinery was shown previously [41,47,48]. Moreover, both of these pathways are involved in VEGF expression [49,50], the key angiogenic factor, whose elevated expression was previously shown to occur after DAA treatment [16,17,18]. Therefore, the contribution of SOF treatment to angiogenesis should be closely examined in the future.

The MAPK cascade was another highly enriched pathway. The MAPK cascade can be activated in an EGFR-dependent manner but also independently of EGFR as a response to various stress stimuli. We confirmed that p38, a member of the MAPK cascade, is indeed activated during SOF treatment and translocated into the nucleus. Interestingly, the role of p38 in the regulation of EGFR in response to stress stimuli was shown by several groups [32,33,51]. However, we could demonstrate that the inhibition of p38 had no effect on the increased activation of EGFR observed during the exposure of cells to SOF, which excludes p38 as a driver of EGFR activation in this context.

Lastly, we explored the possibility of other commonly used NAs exhibiting the same effect on hepatoma cells as SOF. Both AZT and TNF showed a strong effect on the cell cycle; however, only AZT treatment led to increased EGFR expression and its activation. This difference could be explained by the much more pronounced cytotoxic effect of TNF in human cancer cells in comparison to AZT [52], which we also observed in hepatoma cells. AZT is a thymidine analogue able to incorporate into the DNA of host cells, causing a DNA damage response [53]. Additionally, S phase arrest with an increase in Cyclin D1 expression in response to AZT was documented [54]. Interestingly, in the vaginal epithelium of mice, long-term treatment with AZT was correlated with elevated proliferation of the vaginal epithelial basal layer accompanied by the expression of pre-neoplastic markers [55]. Based on our results, the activation of EGFR and its downstream signaling pathway after SOF and AZT treatment could represent a novel putative mechanism of NAs’ toxicity.

Although the molecular mechanisms of HCV-induced hepatocarcinogenesis have not been fully elucidated, in HCV-infected patients, most occurrences of HCC develop only after the establishment of cirrhosis [56]. Therefore, the progression of cirrhosis represents a major risk for HCC development. Several studies implicated the role of overexpression and activation of EGFR in the progression of cirrhosis [57,58]. In fact, elevated levels of EGFR were reported in 68% of HCC and correlated with poor patient outcome [59]. In the context of HCV infection, EGFR is crucial for HCV entry [60]. Moreover, HCV was shown to activate the EGFR during entry and also specifically through its NS3/4A protease during infection [61,62]. Recent research demonstrated an HCV-induced epigenetic signature, which persists after DAA-mediated eradication of HCV. These epigenetic changes were associated with pathways contributing to HCC development [63,64]. Importantly, one of these studies showed that the inhibition of EGFR kinase activity reverted HCV-induced epigenetic signatures [64]. Therefore, EGFR activation seemed to be an important player in facilitating an HCV-induced tumorigenic environment. On this account, we showed that SOF treatment activates EGFR-dependent signaling pathways and thereby could represent an additional factor contributing to the risk of HCC development.

Based on the restricted availability of liver tissue from cirrhotic HCV-infected patients before, during, and after DAA treatment, our study was performed only in the setting of a cell culture system. The limitations of liver biopsies, such as invasiveness, sampling error, and inter-observer variability, are the main reasons why non-invasive technics are preferable for the monitoring of cirrhosis and fibrosis. In this context, the observed drug-induced alteration in the cell phenotype in vitro might not completely recapitulate the in vivo setting. However, we could show an increase in the expression of EGFR and its downstream target B-MYB in freshly isolated primary human hepatocytes, which more closely resemble liver tissue characteristics.

In conclusion, we could show that SOF treatment leads to an increased EGFR-dependent pathway activation, resulting in cell cycle progression, cell survival, and proliferation. Since ongoing activation of the EGFR signaling pathway and its downstream targets is involved in several liver-related pathologic processes, the impact of SOF on them should be further studied.

## Supplementary Materials

The following are available online at https://www.mdpi.com/2073-4409/9/4/1003/s1, Figure S1: Sofosbuvir treatment does not impair mitochondrial respiration, Figure S2: Non-hepatic cells HEK-293 and other DAA do not support SOF-induced phenotype, Figure S3: Application of Erlotinib during SOF treatment induces apoptosis and inhibits proliferation. Figure S4 Activation of EGFR downstream signaling targets after SOF treatment. Tab S1 Predicted activated STK kinases after SOF treatment.
