# Supplementary material for: Sofosbuvir Activates EGFR-Dependent Pathways in Hepatoma Cells with Implications for Liver-Related Pathological Processes
Source: Cells. 2020 Apr 17;9(4):1003. doi: 10.3390/cells9041003 (PMC7225999; doi:10.3390/cells9041003)
Supplement: Supplementary file 1 [file cells-09-01003-s001.pdf]

cells

MDPI

Article

Sofosbuvir Activates EGFR-Dependent Pathways in Hepatoma Cells with Implications for Liver-Related Pathological Processes

Denisa Bojkova [1,2], Sandra Westhaus [1,2], Rui Costa [1], Lejla Timmer [1], Nora Funkenberg [1], Marek Korencak [3], Hendrik Streeck [3], Florian Vondran [4,5], Ruth Broering [6], Stefan Heinrichs [7], Karl S Lang [8] and Sandra Ciesek [1,2,5,*]

[1] Institute of Virology, University Hospital Essen, University Duisburg-Essen, 45147 Essen, Germany; denisa.bojkova@kgu.de (D.B.); sandra.westhaus@kgu.de (S.W.); rcosta@sund.ku.dk (R.C.); lejla.timmer@uk-essen.de (L.T.); nora.funkenberg@googlemail.com (N.F.)

[2] Institute of Medical Virology, University Hospital, Goethe University Frankfurt am Main, 60590 Frankfurt, Germany

[3] Institute for HIV research, University Hospital Essen, University Duisburg-Essen, 45147 Essen, Germany; marek.korencak@uk-essen.de (M.K.); hendrik.streeck@uk-essen.de (H.S.)

[4] Clinic for General, Abdominal and Transplant Surgery, Hannover Medical School, 30625 Hannover, Germany; vondran.florian@mh-hannover.de

[5] German Center for Infection Research (DZIF), 45147 Essen, Germany

[6] Department of Gastroenterology and Hepatology, University Hospital Essen, University Duisburg-Essen, 45147 Essen, Germany; ruth.broering@uni-due.de

[7] Institute for Transfusion Medicine, University Hospital Essen, University Duisburg-Essen, 45147 Essen, Germany; stefan.heinrichs@uk-essen.de

[8] Institute of Immunology, University Hospital Essen, University Duisburg-Essen, 45147 Essen, Germany; KarlSebastian.Lang@uk-essen.de

* Correspondence: sandra.ciesek@kgu.de; Tel.: +49-69-63015219

Received: 9 March 2020; Accepted: 13 April 2020; Published: 17 April 2020

1. Materials and Methods

1.1 Measurement of mitochondrial respiration and the glycolytic function

Cells were cultured with SOF for four consecutive days. At day five, the cells were seeded in a concentration of $4x10^4$ cells/well in XFe 96-well plates (Agilent, Waldbronn, Germany) and let to attach overnight. Mitochondrial respiration was monitored by the oxygen consumption rate (OCR) and glycolysis was controlled by the extracellular acidification rate (ECAR) using a Seahorse XFe Cell Mito Stress Test Kit (Agilent, Waldbronn, Germany) according to the manufacturer´s protocol. The OCR and ECAR values were normalized to the atotal protein amount determined by a Pierce™ BCA Protein Assay Kit (Thermo Fisher Scientific, Schwerte, Germany).

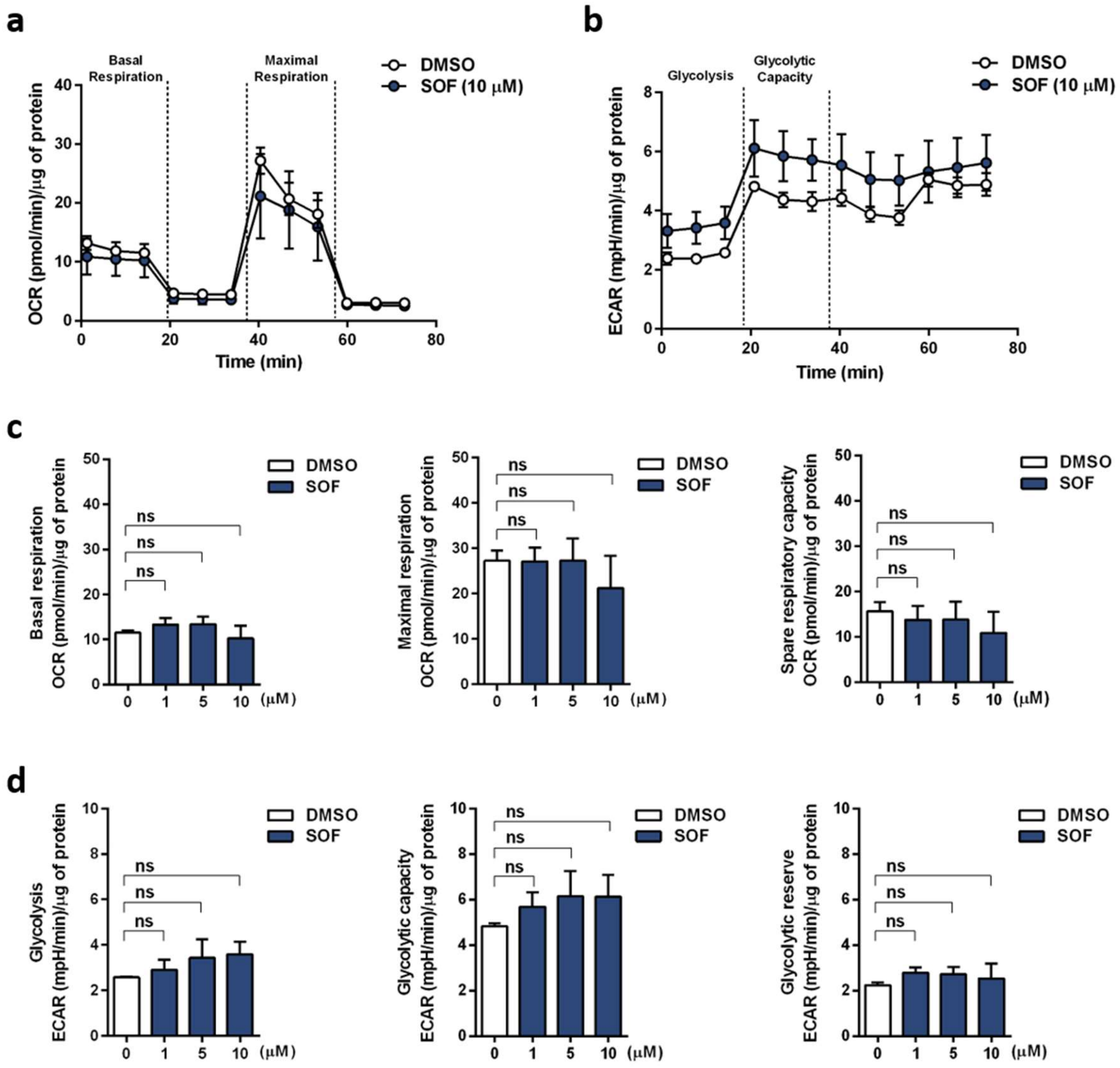

Figure S1. Sofosbuvir treatment does not impair mitochondrial respiration. Cells were cultured with different concentrations of SOF for four consecutive days. At day five, $4x10^4$ cells/well were seeded in XF 96-well plates and mitochondrial respiration and glycolytic function were quantified using a Seahorse extracellular flux analyzer (XFe-96, Agilent). (a, c) Basal respiration, maximal respiration, and spare respiratory capacity were calculated based on the oxygen consumption rate (OCR). (b, d) Glycolysis, glycolytic capacity, and glycolytic reserve were assessed based on the extracellular acidification rate (ECAR). Data are normalized to μg of the total protein amount. Mean + s.d. from three independent experiments are displayed. Statistical significance was determined through one-way ANOVA. ns: not significant.

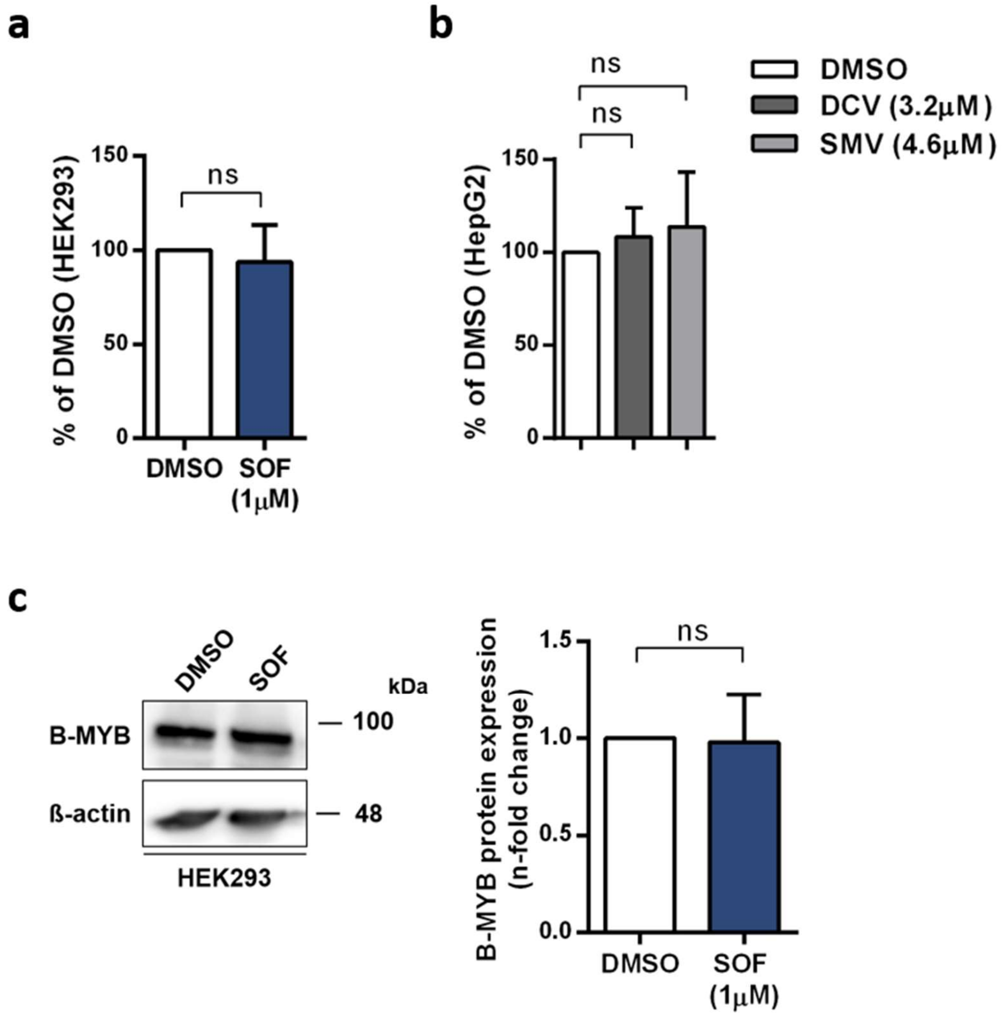

Figure S2. Non-hepatic cells HEK-293 and other DAA do not support an SOF-induced phenotype. (a, b) Proliferation rates of HEK293 cells after SOF treatment (a) and HepG2 cells after DCV or SMV treatment (b) were determined by utilizing trypan blue exclusion. Bar graphs displays results in relation to DMSO. (c) B-MYB protein levels in HEK293 cultured in the presence of SOF were determined by immunoblot analysis. One representative immunoblot is displayed. Relative protein expression is presented as a fold change in relation to the vehicle control DMSO. Displayed data represent mean + s.d. from three independent experiments (a, b) and two independent experiments (c). Statistical significance was determined through an unpaired t-test (a, c) and one-way ANOVA (b). ns: not significant.

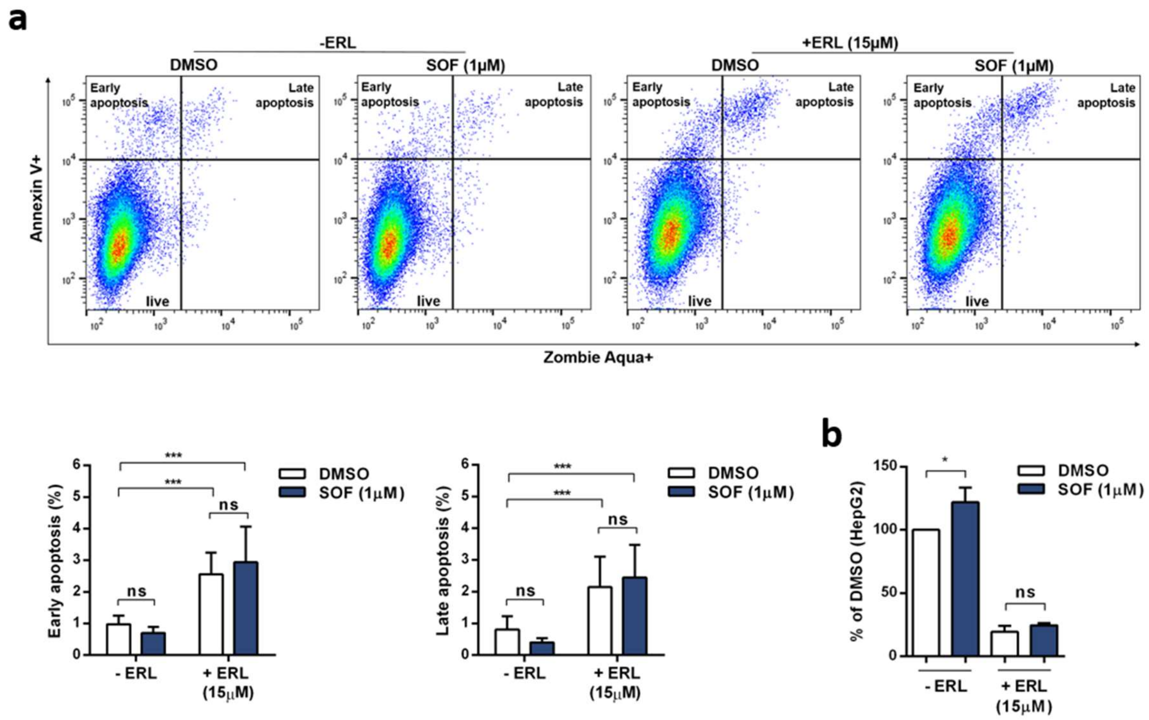

Figure S3. Application of erlotinib during SOF treatment induces apoptosis and inhibits proliferation. HepG2 cells were incubated with ERL and SOF for four consecutive days. At day five, all following analyses were performed. (a) Proportion of apoptotic cells was determined with Annexin V and live/dead cells staining by flow cytometry. Bar graph displays the percentage of cells in early apoptosis and late apoptosis. (b) The proliferation rates were evaluated by trypan blue exclusion and shown as a percentage in relation to the vehicle control DMSO. All experiments are shown as mean + s.d. from three independent experiments. Statistical significance was determined through two-way ANOVA (a, b). ns: not significant; * $p \leq 0.05$; ** $p \leq 0.01$; *** $p \leq 0.005$.

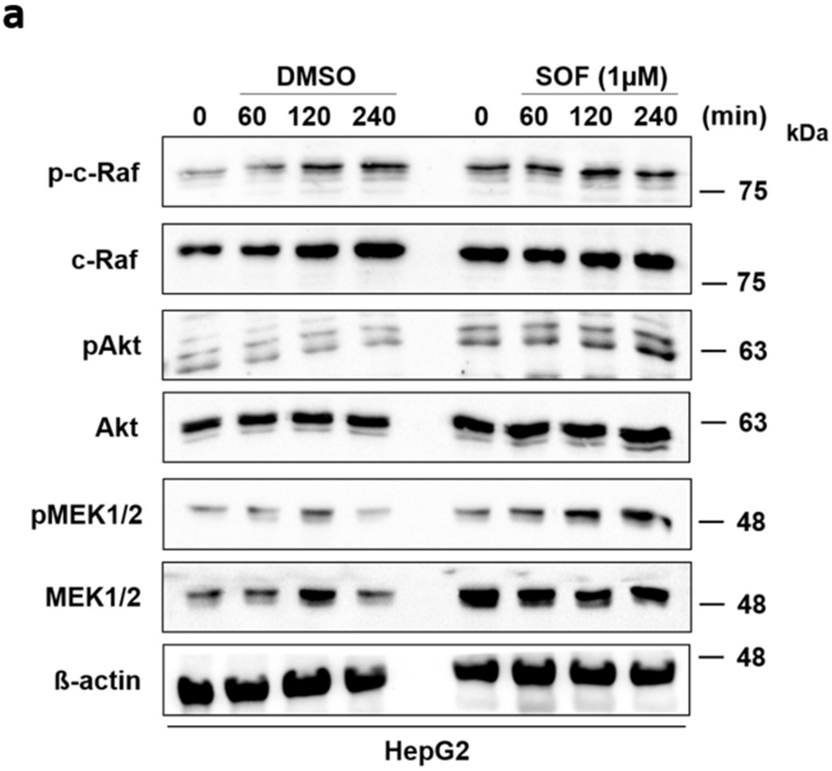

Figure S4. Activation of EGFR downstream signaling targets after SOF treatment. (a) Total protein expression and phosphorylation of target proteins was evaluated by immunoblot analysis. One representative immunoblot is shown.

Table S1. Predicted activated STK kinases after SOF treatment. Based on the comparison of phosphorylated peptides on the array with the databases of documented interactions, such as HPRD, PhosphoSitePlus, as well as the in-silico predictions database PhosphoNET, a specificity and significance score was calculated. The resulting list of kinases is based on the final score, the sum of the median final score, and the median kinase statistics (MKS).

Kinase Name	HepG2	HuH-6	HEK-293
AurB/Aur1	4.374217	3.03169	1.164061
PKD1	4.055393	1.906143	0.359733
RSKL2	3.490924	1.435201	0.035775
IKK[beta]	3.483465	1.730221	0.322747
DAPK2	3.421873	0.252112	0.216946
CaMK2[alpha]	3.346736	1.584383	0.273212
PRKX	3.182962	1.948886	0.088726
CDKL2	3.018253	0.97972	0.814038
PKC[epsilon]	2.875098	2.101545	0.07466
PKA[alpha]	2.856334	2.026203	0.114149
PKC[eta]	2.834951	2.396981	0.056223
IKK[epsilon]	2.788357	1.199546	0.703098
SGK2	2.787967	1.070037	0.187933
PKC[alpha]	2.783897	2.553313	0.129696
PKC[gamma]	2.730528	2.07841	0.052023
PAK1	2.645182	1.851696	0.391424
RSKL1	2.64077	2.990542	0.665425
DAPK3	2.625414	2.804247	0.893947
Akt1/PKB[alpha]	2.619971	1.567764	0.355081
Pim1	2.599516	2.549547	0.788302
CHK2	2.598952	2.640358	1.24863
CaMK4	2.551329	0.967793	0.351173
ERK7	2.519317	2.358516	1.06546
Pim2	2.518634	2.372009	0.890485
PKC[theta]	2.514123	2.181327	0.021492
Pim3	2.486877	2.417713	0.728759
p70S6K[beta]	2.47629	1.824834	0.156769
PKC[delta]	2.468173	1.892108	0.127955
CHK1	2.444178	0.894619	1.44397
PCTAIRE2	2.434013	1.002181	0.227832
MAPKAPK2	2.428079	1.540451	1.421561
TBK1	2.406283	1.295958	0.248604
p38[beta]	2.401599	1.037017	0.126575
ANP[alpha]	2.376676	1.645016	0.306188
SGK1	2.375803	1.982021	0.382716
AurA/Aur2	2.372234	2.23801	1.020769
AMPK[alpha]1	2.3582	1.937989	0.22292
RSK1/p90RSK	2.354727	1.916767	0.262217
JNK2	2.349282	1.046434	0.859831
MSK1	2.318733	2.390597	0.893688
ADCK3	2.308174	1.139974	0.354651

RSK2	2.300832	1.743994	0.587068
CDKL5	2.287687	1.495935	1.22353
PKG1	2.285522	2.2881	0.273141
PKN1/PRK1	2.268168	0.911223	0.004627
Akt2/PKB[beta]	2.253581	1.415509	0.020258
ROCK2	2.220144	1.856898	0.025676
NuaK1	2.215376	2.222923	0.4455
RAF1	2.208523	2.030399	0.371386
BRAF	2.20384	1.39262	0.325423
ATR	2.201845	0.984706	0.704188
PKG2	2.178193	2.71447	0.425434
JNK1	2.146313	1.198105	1.250477
JNK3	2.146313	1.196041	1.235999
MAPKAPK3	2.138058	1.531832	0.396041
RSK3	2.106249	1.518089	0.656172
p70S6K	2.075926	1.819077	0.048804
TNIK/ZC2	2.072682	2.060233	0.923134
PKC[iota]	2.054825	1.065091	0.107054
MAPK14	2.044332	0.875708	0.30967
CK1[alpha]	2.037583	2.375999	0.339512
GSK3[alpha]	2.03709	1.620186	0.370045
ICK	2.027002	1.930407	0.691457
AlphaK1	2.008525	1.915913	1.155768
p38[delta]	1.995322	0.657916	0.072428
DCAMKL1	1.983109	2.736141	0.610241
CDK10	1.88127	3.704281	0.158586
PRKY	1.857864	1.523384	0.155114
CDK9	1.842812	1.682931	1.389388
GSK3[beta]	1.799007	1.960006	0.210386
PKC[beta]	1.760804	2.056166	0.134231
HGK/ZC1	1.750192	1.87224	0.128969
PKC[zeta]	1.702525	1.555805	0.238692
mTOR/FRAP	1.62584	2.778769	0.795234
CDK6	1.578003	0.921009	0.687531
CDK7	1.529946	1.867462	1.072142
ERK5	1.502921	2.7675	1.721496
CDC2/CDK1	1.496093	0.648602	0.882162
CDK4	1.473681	0.99875	0.716556
CDK5	1.462478	1.144918	1.004356
CDK11	1.456666	0.8954	0.834509
PFTAIRE2	1.443057	0.280623	2.180737
p38[gamma]	1.441417	1.957767	0.903558
ERK1	1.430379	3.222845	0.942129
SgK307	1.372155	0.199722	1.269961
CDK2	1.324337	1.958866	1.413946
CDKL1	1.264317	2.85879	0.51856

ROCK1	1.24158	0.988067	0.069619
ERK2	1.170484	2.952321	1.841095
CK1[epsilon]	1.155203	1.23929	0.030355
CDK3	1.130101	1.3681	1.104966
COT	0.368523	2.281348	0.436554

© 2020 by the authors. Licensee MDPI, Basel, Switzerland. This article is an open access article distributed under the terms and conditions of the Creative Commons Attribution (CC BY) license (http://creativecommons.org/licenses/by/4.0/).
